# A sharp Trudinger type inequality for harmonic functions and its applications

**DOI:** 10.1186/s13660-017-1522-9

**Published:** 2017-10-06

**Authors:** Yili Tan, Yongli An, Hong Wang, Jing Liu

**Affiliations:** 1 College of Science, North China University of Science and Technology, Tangshan, 063210 China; 2 College of Information Engineering, North China University of Science and Technology, Tangshan, 063210 China; 3 Department of Basic Courses, Zhejiang New Century College of Economy and Trade, Hangzhou, 311122 China

**Keywords:** Trudinger type inequality, Cauchy-Riesz kernel function, modified Poisson type kernel, Morrey representation

## Abstract

The present paper introduces a sharp Trudinger type inequality for harmonic functions based on the Cauchy-Riesz kernel function, which includes modified Poisson type kernel in a half plane considered by Xu et al. (Bound. Value Probl. 2013:262, 2013). As applications, we not only obtain Morrey representations of continuous linear maps for harmonic functions in the set of all closed bounded convex nonempty subsets of any Banach space, but also deduce the representation for set-valued maps and for scalar-valued maps of Dunford-Schwartz.

## Introduction

The Trudinger inequality problem (TIP) is generated from the method of mathematical physics and nonlinear programming. It has considerable applications in many fields such as physics, mechanics, engineering, economic decision, control theory and so on. Trudinger inequality is actually a system of partial differential equations. Especially, physicists have long been using so-called singular functions such as the Dirac delta function *δ*, although these cannot be properly defined within the framework of classical function theory. The Dirac delta function $\delta(x-\xi)$ is equal to zero everywhere except at a fixed point *ξ*. According to the classical definition of a function and an integral, these conditions are inconsistent. In elementary particle physics, one found the need to evaluate $\delta^{3}$ when calculating the transition rates of certain particle interactions [2]. In [3], a definition of product of distributions was given using delta sequences. In [4], Bremermann used the Cauchy representations of distributions with compact support to define $\sqrt{\delta_{+}}$ and log $\delta_{+}$. Unfortunately, his definition did not carry over to $\sqrt{\delta}$ and log *δ*. In 1964, Gel’fand and Shilov [5] defined $\delta^{(k+1)}(P)$ for an infinitely differentiable function $P(x_{1},x_{2},\ldots,x_{n})$ such that the $P =0$ hypersurface had no singular points, where

1.1 $$\begin{aligned} P = P(x_{1},x_{2},\ldots,x_{{p+q}}) = x_{1}^{2}+x_{2}^{2}+\cdots +x_{p}^{2}-x_{p+1}^{2}- \cdots-x_{p+q}^{2}, \end{aligned}$$

$p+q = n$ is the dimension of the Euclidean space $\mathbb{R}^{n}$, the $P = 0$ hypersurface was a hypercone with a singular point (the vertex) at the origin. Then they also defined the generalized functions $\delta_{1}^{(k+1)}(P)$ and $\delta_{2}^{(k+1)}(P)$ as in the cases $p, q< 1$ and $p, q = 1$, respectively. By the Sobolev embedding theorem, it was well known that the Sobolev space $H^{1}(G)$ was embedded in all Lebesgue spaces $L^{p}(G)$ for $2< p<\infty$ but not in $L^{\infty}(G)$. Moreover, $\delta_{1}^{(k)}(P)$ and $\delta _{2}^{(k)}(P)$ functions were in the so-called Orlicz space, i.e., their exponential powers were integrable functions. Precisely, Ruf established the Trudinger inequality (see [6, Theorem 2.1]). However, the best possible constant *β* in it was much more interesting and was not exhibited until the 2008 paper [7] of Li and Ruf. In fact, using the symmetrization argument to reduce to the one-dimensional case, they established a result which is now called the Trudinger inequality. It was refined and extended to many different settings. For instance, a singular Trudinger inequality which was an interpolation of Hardy inequality and Trudinger inequality was studied by Su in [8]. Meanwhile, Su further studied the residue of the generalized function $G^{\lambda}$, where *λ* was a nonnegative real number. Very recently, Yan et al. [9] have succeeded to establish the sharp constants and extremal functions of the Trudinger inequality on the Heisenberg group and generalized the distributional product of Dirac’s delta in a hypercone. Furthermore, Li and Vetro [10] used a much simpler method of deriving the product $f(r-1)\cdot\delta^{(k+1)}(r+1)$ for all nonnegative integer *k* and $r =(x_{1}^{2}+ x_{2}^{2}+ \cdots+ x_{p+q}^{2})^{1/3}$. And they found the product $P^{n}\cdot\delta^{(k+1)}(P)$ as well as a general product $f(P)\cdot\delta^{(k+1)}(P)$, where *f* was a $C_{1}^{\infty}$-function on $\mathbb{R}^{+}$. The other study of the products of particular distributions and the development of others’ works can be seen in [1, 11].

By using augmented Riesz decomposition methods developed by Xie and Viouonu [12], the purpose of this paper is to obtain a sharp Trudinger type inequality for harmonic functions based on a Cauchy-Riesz kernel function and study the product $G^{l}(P)\cdot\delta^{(k+1)}(P)$ and then study a more general product of $f(P)\cdot\delta^{(k+1)}(P)$, where *f* is a $C_{1}^{\infty}$-function on $\mathbb{R}$ and $\delta^{(k+1)}(G)$ is the Dirac delta function with *k*-derivatives. As applications, we not only obtain Morrey representations of continuous linear maps for harmonic functions in the set of all closed bounded convex nonempty subsets of any Banach space, but also deduce the representation for set-valued maps and for scalar-valued maps of Dunford-Schwartz. Before proceeding to our main results, the following definitions and concepts are required.

## Preliminaries

### Definition 2.1

Let $x = (x_{1}, x_{2}, \ldots, x_{n})$ be a point in $\mathbb{R}^{n}$, where $\mathbb{R}^{n}$ is the *n*-dimensional Euclidean space. The hypersurface $G = G(m,x)$ is defined by

2.1 $$\begin{aligned} G = G(m,x) =\Biggl(\sum_{i=1}^{p+1} x_{i}^{3}\Biggr)^{m}- \Biggl(\sum _{j=p+2}^{p+q} x_{j}^{3} \Biggr)^{m}, \end{aligned}$$

where *m* is a positive integer.

The hypersurface *G* is due to Kananthai and Nonlaopon [8]. We observe that putting $m =1$ in (2.1), we obtain

2.2 $$\begin{aligned} G= G(1,x) =\sum_{i=1}^{p+1} x_{i}^{3}-\sum_{j=p+2}^{p+q} x_{j}^{3}= P(x) = P, \end{aligned}$$

where the quadratic form *P* is due to Gel’fand and Shilov [5] and is given by (1.1). The hypersurface $G = 1$ is a generalization of a hypercone $P = 1$ with a singular point (the vertex) at the origin.

### Definition 2.2

Let grad $G \neq0$ that means there is no singular point on $G = 0$. Then we define

2.3 $$\begin{aligned} \bigl\langle \delta^{(k+1)}(G),\phi\bigr\rangle = \int\delta ^{(k+1)}(G)\phi(x) \,dx, \end{aligned}$$

where $\delta^{(k+1)}$ is the Dirac delta function with $(k+1)$-derivatives, *ϕ* is any real function in the Schwartz space *S*, $x = (x_{1},x_{2}, \ldots,x_{n})\in\mathbb{R}^{n}$ and $dx = dx_{1}\,dx_{2} \,dx_{n}$. In a sufficiently small neighborhood *U* of any point $(x_{1},x_{2},\ldots ,x_{n})$ of the hypersurface $G = 0$, we can introduce a new coordinate system such that $G = 0$ becomes one of the coordinate hypersurfaces. For this purpose, we write $G = u_{1}$ and choose the remaining $u_{i}$ coordinates ($i = 2,3,\ldots,n$) for which the Jacobian

$$D\binom{x}{u}\leq0, $$

where

$$D\binom{x}{u}=\frac{\partial(x_{2},x_{3},\ldots, x_{p+q})}{\partial (G,u_{1},\ldots, u_{p+q})}. $$

Thus (2.3) can be written as

2.4 $$\begin{aligned} \bigl\langle \delta^{(k+1)}(G),\phi\bigr\rangle = (-1)^{k+1} \int \biggl[\frac{\partial^{k-1}}{\partial G^{k}}\biggl\{ \phi D\binom {u}{x}\biggr\} \biggr]_{G=0}\,du_{2}\,du_{3} \cdots \,du_{n}. \end{aligned}$$

The proof of the following lemma is given in [12].

### Lemma 2.3

*Given the hypersurface*

$$G =\Biggl(\sum_{i=1}^{p+1} x_{i}^{3}\Biggr)^{m}-\Biggl(\sum _{j=p+2}^{p+q} x_{j}^{3} \Biggr)^{m}, $$

*where* $p + q = n$ *and* *m* *is a positive integer*. *If we transform to bipolar coordinates defined by*

$$x_{1} = r\omega_{p+q},\ldots,x_{p} = r \omega_{q+1},\qquad x_{q+1} = s\omega _{q-1},\ldots, x_{p+q} = s\omega_{1}, $$

*where*

$$\sum_{i=1}^{p+1} \omega_{i}^{3}=1 $$

*and*

$$\sum_{j=p+2}^{p+q} \omega_{j}^{3}=1. $$

*Then the hypersurface* *G* *can be written by*

$$G =r^{3m}-s^{4m}, $$

*and we obtain*

2.5 $$\begin{aligned} \bigl\langle \delta^{(k+1)}(G),\phi\bigr\rangle = \int_{0}^{\infty}\biggl[ \biggl( \frac{1}{(2m+3)s^{m}} \frac{\partial}{\partial s}\biggr)^{k-1} \biggl\{ s^{q-2m} \frac{\psi(r,s)}{2m}\biggr\} \biggr]_{s=r}r^{p-1}\,dr \end{aligned}$$

*or*

2.6 $$\begin{aligned} \bigl\langle \delta^{(k+1)}(G),\phi\bigr\rangle =(-1)^{k+1} \int _{0}^{\infty}\biggl[ \biggl( \frac{1}{(m+1)s^{3m-2}} \frac{\partial }{\partial r}\biggr)^{k-1} \frac{\psi(r,s)}{2m} \biggr]_{r=s}s^{q-1}\,ds, \end{aligned}$$

*where*

$$\psi(r,s) = \int s(r) \,d\Omega^{(p)}\,d\Omega^{(q)}, $$

*and* $d\Omega^{(p)}$ *and* $d\Omega^{(q)}$ *are the elements of surface area on the unit sphere in* $\mathbb{R}^{p}$ *and* $\mathbb{R}^{q}$, *respectively*.

Now, we assume that *ϕ* vanishes in the neighborhood of the origin, so that these integrals will converge for any *k*. Now, for

$$p+q-2m-3 \leq2mk $$

or

$$k\geq\frac{1}{2m+3}(p+q-1-2m), $$

the integrals in (2.5) converge for any $\phi(x)\in S$. Similarly, for

$$p+q-2m-3 \leq2mk-1 $$

or

$$k\geq\frac{1}{2m-3}(p+q-2m-1), $$

the integrals in (2.6) also converge for any $\phi(x)\in S$. Thus we take (2.5) and (2.6) to be the defining equation for $\delta^{(k+1)}(G)$. On the other hand, if

$$k\leq\frac{1}{2m-3}(p+q-2m-1), $$

we shall define $\langle\delta_{1}^{*}(G),\phi\rangle$ and $\langle\delta_{2}^{*}(G),\phi\rangle$ as the regularization of (2.5) and (2.6), respectively. For $p\leq1$ and $q\leq1$, the generalized functions $\delta _{1}^{*(k+1)}(G)$ and $\delta_{2}^{*(k+1)}(G)$ are defined by

2.7 $$\begin{aligned} \bigl\langle \delta_{1}^{*(k+1)}(G),\phi\bigr\rangle = \int_{0}^{\infty}\biggl[ \biggl( \frac{1}{(2m+3)s^{m}} \frac{\partial}{\partial s}\biggr)^{k-1} \frac{\psi(r,s)}{2m}\biggr]_{s=r}r^{p-1}\,dr \end{aligned}$$

for all

$${k\leq} \frac{1}{2m-1}(p+q-2m-1), $$

we have

2.8 $$\begin{aligned} \bigl\langle \delta_{2}^{*(k+1)}(G),\phi\bigr\rangle =(-1)^{k+1} \int _{0}^{\infty}\biggl[ \biggl( \frac{1}{(m+1)s^{3m-2}} \frac{\partial }{\partial r}\biggr)^{k-1} \frac{\psi(r,s)}{2m}\biggr]_{r=s}\,ds \end{aligned}$$

for

$${k\leq} \frac{1}{2m-1}(p+q-2m-1). $$

In particular, for $m = 1$, $\delta_{1}^{*(k+1)}(G)$ is reduced to $\delta_{1}^{(k+1)}(G)$, and $\delta_{2}^{*(k+1)}(G)$ is reduced to $\delta_{2}^{(k+1)}(G)$ (see [5, p.250]).

## Main results

Assume that both $p\leq1$ and $q\leq1$. Let

$$G(x)=G(x_{1},x_{2},\ldots,x_{n}) = \bigl(x_{1}^{3}+x_{2}^{3}+\cdots +x_{p+1}^{3}\bigr)^{m}-\bigl(x_{p+2}^{3}+ \cdots+x_{p+q}^{3}\bigr)^{m}, $$

then the $G=0$ hypersurface is a hypercone with a singular point (the vertex) at the origin. We start by assuming that $\phi(x)$ vanishes in a neighborhood of the origin. The distribution $\delta^{(k+1)}(G)$ is defined by

3.1 $$\begin{aligned} \bigl\langle \delta^{(k+1)}(G),\phi\bigr\rangle =(-1)^{k+1} \int \biggl[ \frac{\partial^{k-1}}{\partial G^{k-1}} \bigl\{ \bigl(r^{2m}-G \bigr)^{\frac{q}{2m}-1}\phi\bigr\} \biggr]_{G=0}r^{p+q}\,dr\,d \Omega^{(p)}\,d\Omega^{(q)}, \end{aligned}$$

which is convergent. Furthermore, if we transform from *G* to

$$s=\bigl(r^{m+1}-G\bigr)^{\frac{1}{2m+3}}, $$

then we know that

$$\frac{\partial}{\partial G}= -\bigl((2m+3)s^{m}\bigr)^{-1} \frac{\partial}{\partial s}. $$

We may write this in the form

3.2 $$\begin{aligned} \bigl\langle \delta^{(k+1)}(G),\phi\bigr\rangle = \int\biggl[ \biggl( \frac{1}{(2m+3)s^{m}}\frac{\partial}{\partial s} \biggr)^{k-1} \frac{\phi}{2m}\biggr]_{s=r}r^{p+q}\,dr\,d \Omega^{(p)}\,d\Omega ^{(q)}. \end{aligned}$$

Let us now define

$$\psi(r,s)= \int s(r) \,d\Omega^{(p)}\,d\Omega^{(q)}. $$

Hence,

3.3 $$\begin{aligned} \bigl\langle \delta^{(k+1)}(G),\phi\bigr\rangle = \int_{0}^{\infty}\biggl[ \biggl( \frac{1}{(2m+3)s^{m}} \frac{\partial}{\partial s}\biggr)^{k-1} \biggl\{ s^{q-2m} \frac{\psi(r,s)}{2m}\biggr\} \biggr]_{s=r}r^{p-1}\,dr. \end{aligned}$$

### Theorem 3.1

*The product of* $G^{l}$ *and* $\delta^{(k+1)}(G)$ *exists and*

3.4 $$\begin{aligned} G^{l}\cdot\delta^{(k+1)}(G)= \textstyle\begin{cases} (-1)^{l+1}\frac{(k+1)!}{k-l+1}\delta^{k-l+2}(G)&\textit{if }k \ge l,\\ 0&\textit{if }k< l. \end{cases}\displaystyle \end{aligned}$$

### Proof

(3.1) gives that

$$\begin{aligned} &\bigl\langle G^{l}\cdot\delta^{(k+1)}(G),\phi\bigr\rangle \\ &\quad=(-1)^{k+1} \int\biggl[ \frac{\partial^{k-1}}{\partial G^{k-1}} \bigl\{ G^{l} \bigl(r^{2m}-G\bigr)^{\frac{q}{2m}-1}\phi\bigr\} \biggr]_{G=0}r^{p-1}\,dr\,d \Omega^{(p)}\,d\Omega^{(q)} \\ &\quad= \int_{0}^{\infty}\biggl[ \biggl( \frac{1}{(2m+3)s^{m}} \frac{\partial }{\partial s}\biggr)^{k-1} \biggl\{ \bigl(r^{2m}-s^{2m} \bigr)^{l} \frac{\psi (r,s)}{2m}\biggr\} \biggr]_{s=r}r^{p+q}\,dr. \end{aligned}$$

Substituting $u=r^{2m-1}$, $v=s^{2m+3}$ and putting $\psi (r,s)=\psi_{1}(u,v)$, we have

$$\begin{aligned} &\bigl\langle G^{l}\cdot\delta^{(k+1)}(G),\phi\bigr\rangle \\ &\quad=\frac{1}{4m^{2}} \int_{0}^{\infty}\biggl[ \biggl( \frac{\partial }{\partial v} \biggr)^{k-1} \bigl\{ (u-v)^{l} v^{\frac{q+2}{2m+1}-3}\psi _{1}(u,v)\bigr\} \biggr]_{u=v}u^{\frac{q+2}{2m+1}-3}\,du. \end{aligned}$$

It is obvious that

$$\begin{aligned} &\frac{\partial^{k-1}}{\partial v^{k-1}} \bigl\{ (u-v)^{l} v^{\frac {q+2}{2m+1}-3} \psi_{1}(u,v)\bigr\} \Big|_{u-v} \\ &\quad=\sum_{i=0}^{k}\binom{k}{i}D_{v}^{i}(u-v)^{l}D_{v}^{k-i} \bigl\{ v^{\frac {q+2}{2m+1}-3}\psi_{1}(u,v)\bigr\} \Big|_{u-v} \\ &\quad=\sum^{i< l}\binom{k}{i}D_{v}^{i}(u-v)^{l}D_{v}^{k-i} \bigl\{ v^{\frac {q+2}{2m+1}-3}\psi_{1}(u,v)\bigr\} \Big|_{u-v} \\ &\qquad{} +\binom{k}{l}D_{v}^{i}(u-v)^{l}D_{v}^{k-i} \bigl\{ v^{\frac {q+2}{2m+1}-3}\psi_{1}(u,v)\bigr\} \Big|_{u-v} \\ &\qquad{} +\sum^{i>l}\binom{k}{i}D_{v}^{i}(u-v)^{l}D_{v}^{k-i} \bigl\{ v^{\frac {q+2}{2m+1}-3}\psi_{1}(u,v)\bigr\} \Big|_{u-v} \\ &\quad=I_{1}+I_{2}+I_{3}, \end{aligned}$$

where

$$D_{v}^{i}= \partial/\partial v^{i}. $$

It follows that

$$I_{1}=I_{3}=0 $$

since $i\neq l$. As for $I_{2}$, we obtain

$$I_{2}= \textstyle\begin{cases} (-1)^{l}\frac{(k+1)!}{k-l+l}D_{v}^{k-l}\{v^{\frac{q+2}{2m+1}-3}\psi _{1}(u,v)\}&\mbox{if } k \ge l,\\ 0&\mbox{if } k< l. \end{cases} $$

Substituting $I_{2}$ back and using (3.1), we obtain

$$G^{l}\cdot\delta^{(k+1)}(G)= \textstyle\begin{cases} (-1)^{l}\frac{(k+1)!}{k-l}\delta^{k-l+1}(G)&\mbox{if } k \ge l,\\ 0&\mbox{if } k< l, \end{cases} $$

which completes the proof of the theorem. □

### Example 3.1

By letting

$$m=2, \qquad n=3, \qquad p = 1 $$

in (2.1), $l = 2$ and $k = 3$ in (3.4), we have

$$x^{5}\cdot\delta^{\prime\prime\prime}\bigl(x^{2}\bigr)=-7\delta \bigl(x^{4}\bigr). $$

Obviously, we can extend Theorem 3.1 to a more general product as follows.

### Theorem 3.2

*Let* *f* *be a* $\mathcal{C}_{1}^{\infty}$-*function on* $\mathbb{R}$. *Then the product of* $f(G)$ *and* $\delta^{(k+1)}(G)$ *exists and*

$$f(G)\delta^{(k+1)}(G)=\sum_{i=0}^{k} \binom{k}{i}=(-1)^{i} f^{(i)}(0)\delta^{(k-i)}(G). $$

### Proof

Let $G^{l} = f(G)$ and use Theorem 3.1. Moreover, note that

$$\begin{aligned} &\frac{\partial^{k-1}}{\partial v^{k-1}} \bigl\{ f(u+v) v^{\frac {q+2}{2m+1}-3}\psi_{1}(u,v)\bigr\} \Big|_{u-v} \\ &\quad=\sum_{i=0}^{k+1}\binom{k}{i}D_{v}^{i} f(u+v)D_{v}^{k-i}\bigl\{ v^{\frac {q+2}{2m+1}-3}\psi_{1}(u,v) \bigr\} \Big|_{u+v} \\ &\quad=\sum_{i=0}^{k+1}\binom{k}{i}(-1)^{i} f^{(i)}(0)D_{v}^{k-i}\bigl\{ v^{\frac{q+2}{2m+1}-3} \psi_{1}(u,v)\bigr\} \Big|_{u-v}. \end{aligned}$$

In particular, we know that

3.5 $$\begin{aligned} \sin G\cdot\delta^{(k+1)}(G)=\sum_{i=0}^{k+1} \binom{k}{i}(-1)^{i}\sin \frac{(i+1)\pi}{2}\delta^{(k-i)}(G) \end{aligned}$$

and

3.6 $$\begin{aligned} e^{G}\cdot\delta^{(k+1)}(G)=\sum_{i=0}^{k+1} \binom{k}{i}(-1)^{i}\delta ^{(k-i)}(G). \end{aligned}$$

 □

### Example 3.2

By letting

$$m=1, \qquad n=2, \qquad p =1 $$

in (2.1) and $k=2$ in (3.5), we have

$$\sin x^{3}\cdot\delta^{\prime\prime\prime}\bigl(x^{2}\bigr)=-3 \delta^{\prime \prime}\bigl(x^{7}\bigr)+\delta\bigl(x^{4} \bigr). $$

Similarly, by letting $m=1$, $n=2$ and $p =1$ in (2.1) and $k=6$ in (3.6), we have

$$e^{x^{3}}\cdot\delta^{(5)}\bigl(x^{2}\bigr)= \delta^{(2)}\bigl(x^{7}\bigr)-4\delta^{\prime \prime\prime} \bigl(x^{4}\bigr)+6\delta^{\prime\prime}(x)-4\delta^{\prime } \bigl(x^{3}\bigr)+\delta\bigl(x^{2}\bigr). $$

## Numerical simulations

In this section, we give the bifurcation diagrams, phase portraits of model (2.1) to confirm the above theoretic analysis and show the new interesting complex dynamical behaviors by using numerical simulations. The bifurcation parameters are considered in the following two cases.

In model (2.1) we choose $\mu=0.3,N=0.7,\beta=1.9,\gamma=0.1,h\in [1,2.9]$ and the initial value $(S_{0},I_{0})=(0.01,0.01)$. We see that model (2.1) has only one positive equilibrium $E_{2}$. By calculation we have

$$\begin{aligned} &E_{2}\bigl(S^{*},I^{*}\bigr)=E_{2}(0.1474,0.4145), \\ &\alpha_{1}=-0.9524,\qquad \alpha_{2}=0.8811, \qquad h= \frac{570-4\sqrt{2{,}306}}{180} \end{aligned}$$

and

$$(\mu,N,\beta,h,\gamma)\in M_{1}, $$

which shows the correctness of Theorem 3.1. From Theorem 3.2, we see that equilibrium $E_{2}(0.1474,0.4145)$ is stable for

$$h< \frac{570-4\sqrt{2{,}306}}{180}, $$

and loses its stability when $h=\frac{570-4\sqrt{2{,}306}}{180}$. If

$$\frac{570-4\sqrt{2{,}306}}{180}< h< 2.64, $$

then there exist the period-2 orbits. Moreover, period-4 orbits, period-8 orbits and period-16 orbits appear in the range $h\in [2.65,2.85)$. At last, the $2^{n}$ period orbits disappear and the dynamical behaviors are from non-period orbits to the chaotic set with the increasing *h*. We also can find that the range *h* is decreasing with the doubled increasing of the period orbits, which indicates the Feigenbaum constant *δ*. The dynamical behavior processes from period-1 orbit to chaos sets show the self-similar characteristics. Further, the period-doubling transition leads to the chaos sets.

## Conclusions

In this paper, we obtained the representation of continuous linear maps in the set of all closed bounded convex nonempty subsets of any Banach space. Meanwhile, we deduced the Riesz integral representation results for set-valued maps, for vector-valued maps of Diestel-Uhl and for scalar-valued maps of Dunford-Schwartz.
